# Daniel T.L. Shek: Pioneer in Chinese Quality of Life Research

**DOI:** 10.1007/s11482-018-9597-0

**Published:** 2018-02-07

**Authors:** Daniel Shek

**Affiliations:** Department of Applied Social Sciences, The Hong Kong Polytechnic University, Hung Hom, Kowloon, Hong Kong


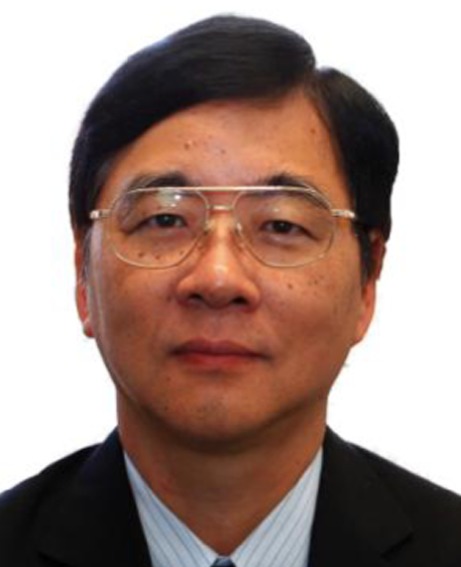


Daniel Shek was born in Hong Kong. He has one elder sister and four younger sisters. His father died when he was in Grade 8. He obtained his bachelor’s degree in Psychology in 1979 at The University of Hong Kong (HKU). He then worked as a full-time demonstrator and pursued his full-time PhD study at HKU for four years.

After he obtained his PhD degree, he taught at City Polytechnic of Hong Kong from 1984 to 1987. He then moved to The Chinese University of Hong Kong (CUHK) teaching social work for 22 years. In response to the severe lack of studies on quality of life of Chinese people, he has gradually developed his research programs on the well-being of Chinese adolescents and families since 1980s.

Daniel Shek is currently Associate Vice President (Undergraduate Programme) and Chair Professor of Applied Social Sciences at The Hong Kong Polytechnic University (PolyU). He is also Advisory Professor of East China Normal University, Honorary Professor of Kiang Wu Nursing College of Macau, and Adjunct Professor of University of Kentucky College of Medicine. To promote holistic development of Chinese university students, he has helped to develop the leadership and service learning components of the new 4-year undergraduate curriculum at PolyU.

Professor Shek is a psychologist with research interests in positive youth development, family process, scale development, quality of life, program evaluation, addiction, spirituality, leadership and university social responsibility. He has conducted extensive research on the well-being of Chinese adolescents and Chinese families. In the past decade, he has been leading a research project entitled “P.A.T.H.S. to Adulthood: A Jockey Club Youth Enhancement Scheme” (Project P.A.T.H.S.) which is financially supported by The Hong Kong Jockey Club Charities Trust. In this project, he worked together with his colleagues to develop a positive youth development program for junior secondary school students in Hong Kong, trained potential program implementers, provided support in program implementation and conducted comprehensive evaluation of the program. Because of the overwhelming success of the project, the program has been transplanted to mainland China with the financial support of Tin Ka Ping Foundation.

Daniel Shek is Chief Editor of *Journal of Youth Studies* and *Applied Research in Quality of Life*, Associate Editor of *Frontiers in Child Health and Human Development* and past Consulting Editor of *Journal of Clinical Psychology*. He is an Editorial Board member of *Encyclopedia of Quality of Life and Well-Being Research* published by Springer, Series Editor of *The Quality of Life in Asia* published by Springer and an Associate Editor of *The Encyclopedia of Family Studies* published by Wiley-Blackwell. He is an Editorial Advisor of *The British Journal of Social Work* and Editorial Board member of several international journals, including *Social Indicators Research*, *Journal of Adolescent Health*, *International Journal of Behavioral Development,* and *Journal of Child and Family Studies.*

Professor Shek has to date published more than 600 articles in international refereed journals. His research project on the adjustment of Chinese midlife people has been rated “excellent” by the Research Grants Council, Hong Kong. He was awarded the CUHK Research Excellence Award 2007 by The Chinese University of Hong Kong. In addition to his research interests, Professor Shek is passionate about teaching. He was awarded two teaching awards during his stay at CUHK. In 2016, he was awarded the Bronze Award in the category of “Ethical Leadership” and the Bronze Award in the category of “Social Enterprise” at the QS Reimagine Education Awards. In 2017, he was further awarded the Silver Award under the category of “Ethical Leadership” and the Silver Award under the category of “Sustainability” in the QS Reimagine Education Awards.

Professor Shek has served on many high-level governmental advisory committees in the Hong Kong Government, including the Action Committee Against Narcotics, Commission on Youth, Women’s Commission, Fight Crime Committee, Beat Drugs Fund Association, Independent Commission Against Corruption, Mental Health Review Tribunal, and Committee on Child Fatality. Professor Shek served as the past Chairman of the Action Committee Against Narcotics (2009–2014) and is currently Chairman of the Family Council, Government of the Hong Kong Special Administrative Region. He has also been Chairman of Heep Hong Society and Society of Boys’ Centres. He was awarded the Bronze Bauhinia Star and Silver Bauhinia Star by the Government of the Hong Kong Special Administrative Region in 2000 and 2013, respectively. He is also a Non-official Justice of the Peace in Hong Kong.

Daniel Shek lives in Hong Kong with his wife, Jennifer Lee, and their two children, Moses and Esther.
